# Supplementary material for: Proteomic and metabolomic analyses of the human adult myocardium reveal ventricle-specific regulation in end-stage cardiomyopathies
Source: Commun Biol. 2024 Dec 19;7:1666. doi: 10.1038/s42003-024-07306-y (PMC11659555; doi:10.1038/s42003-024-07306-y)

Supplementary Figure 1

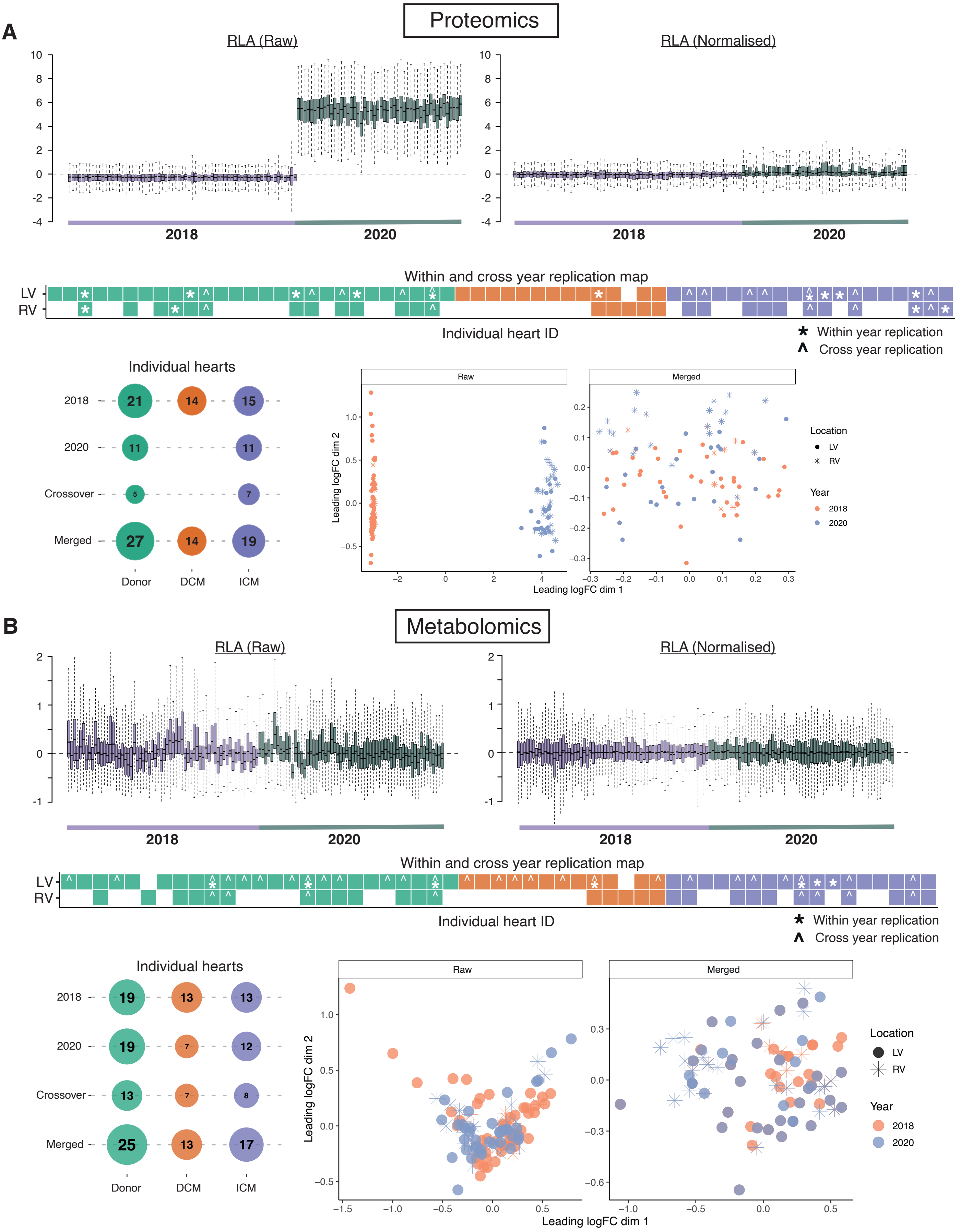

Supplementary Figure 2

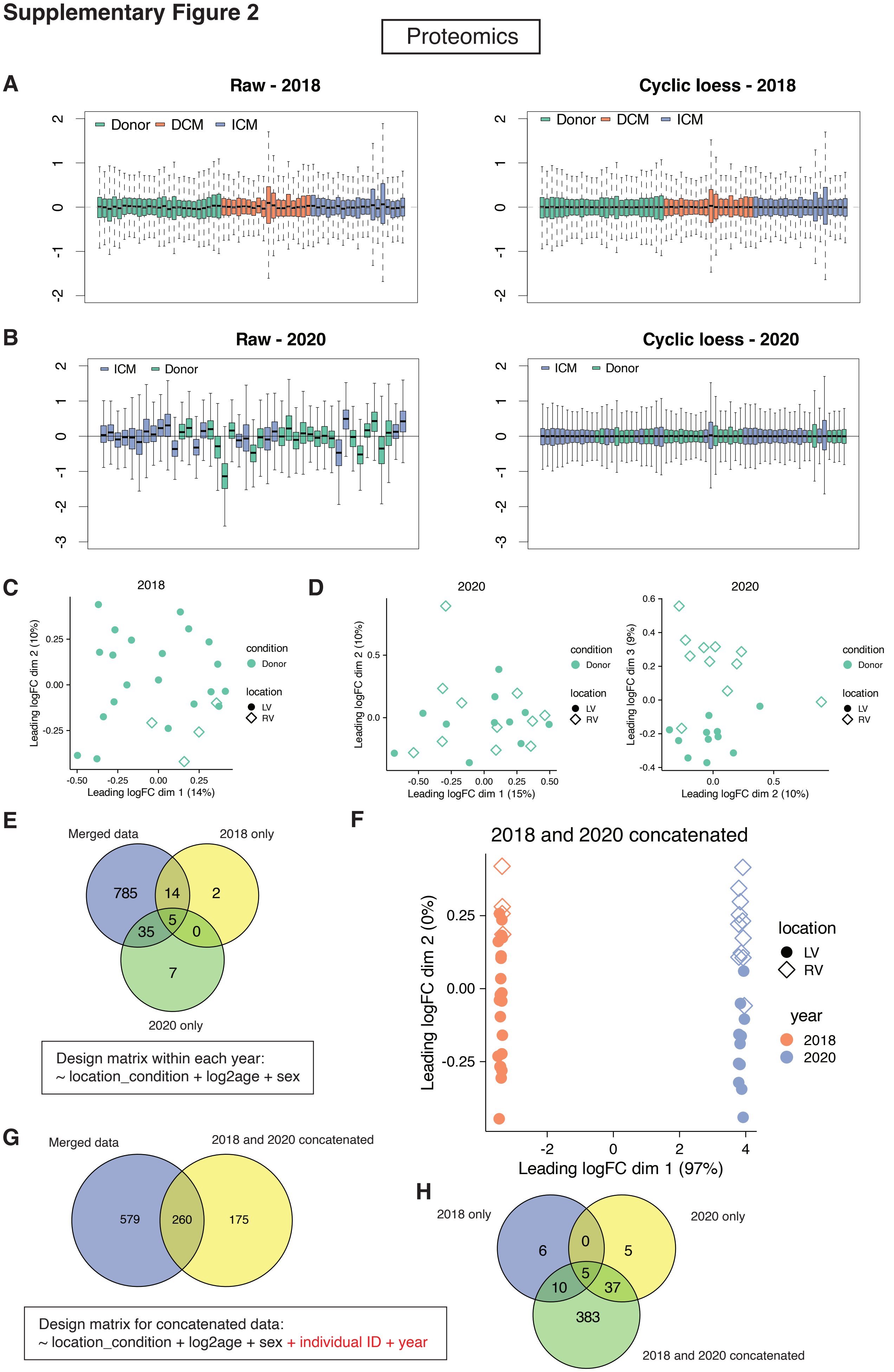

Supplementary Figure 3

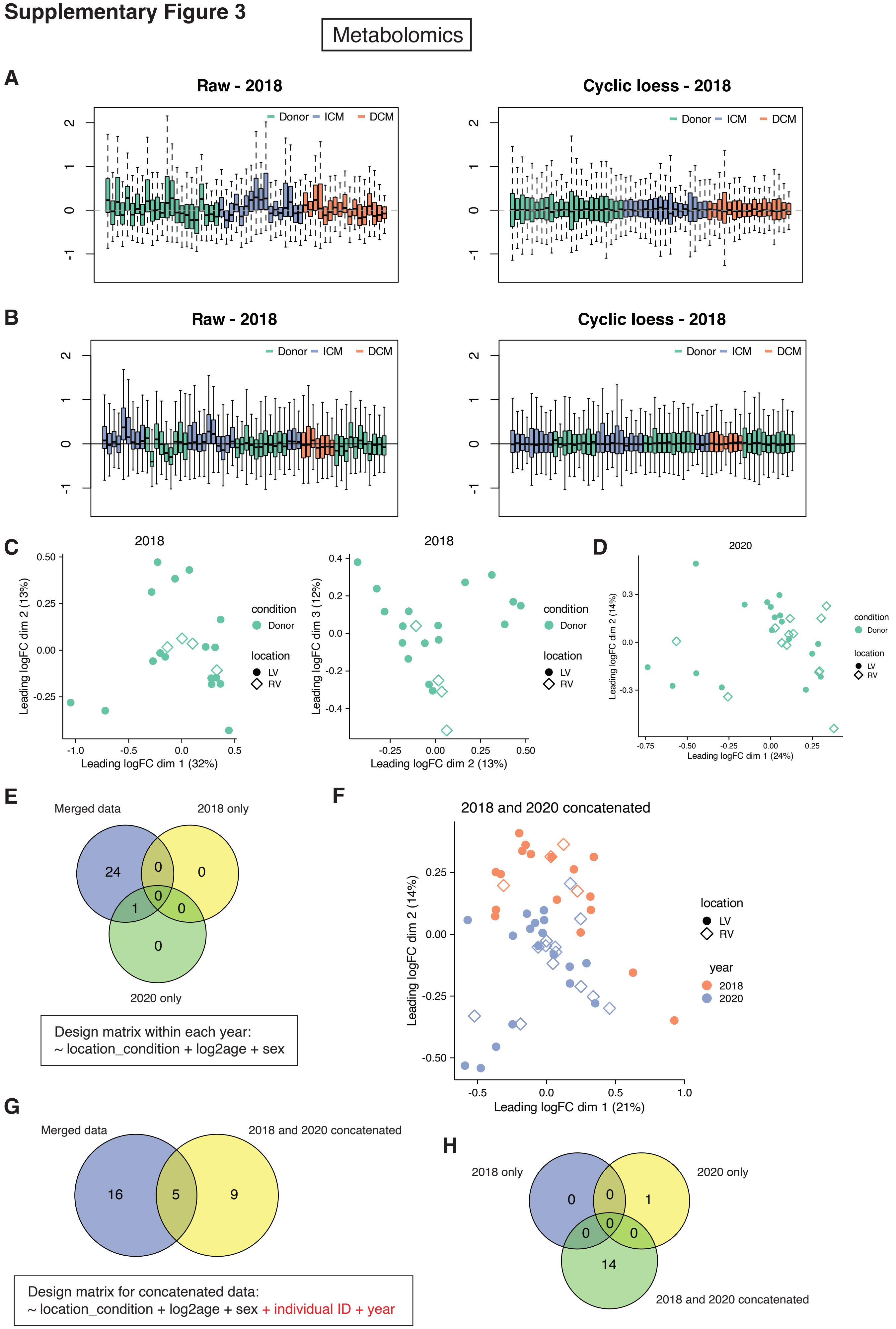

Supplementary Figure 4

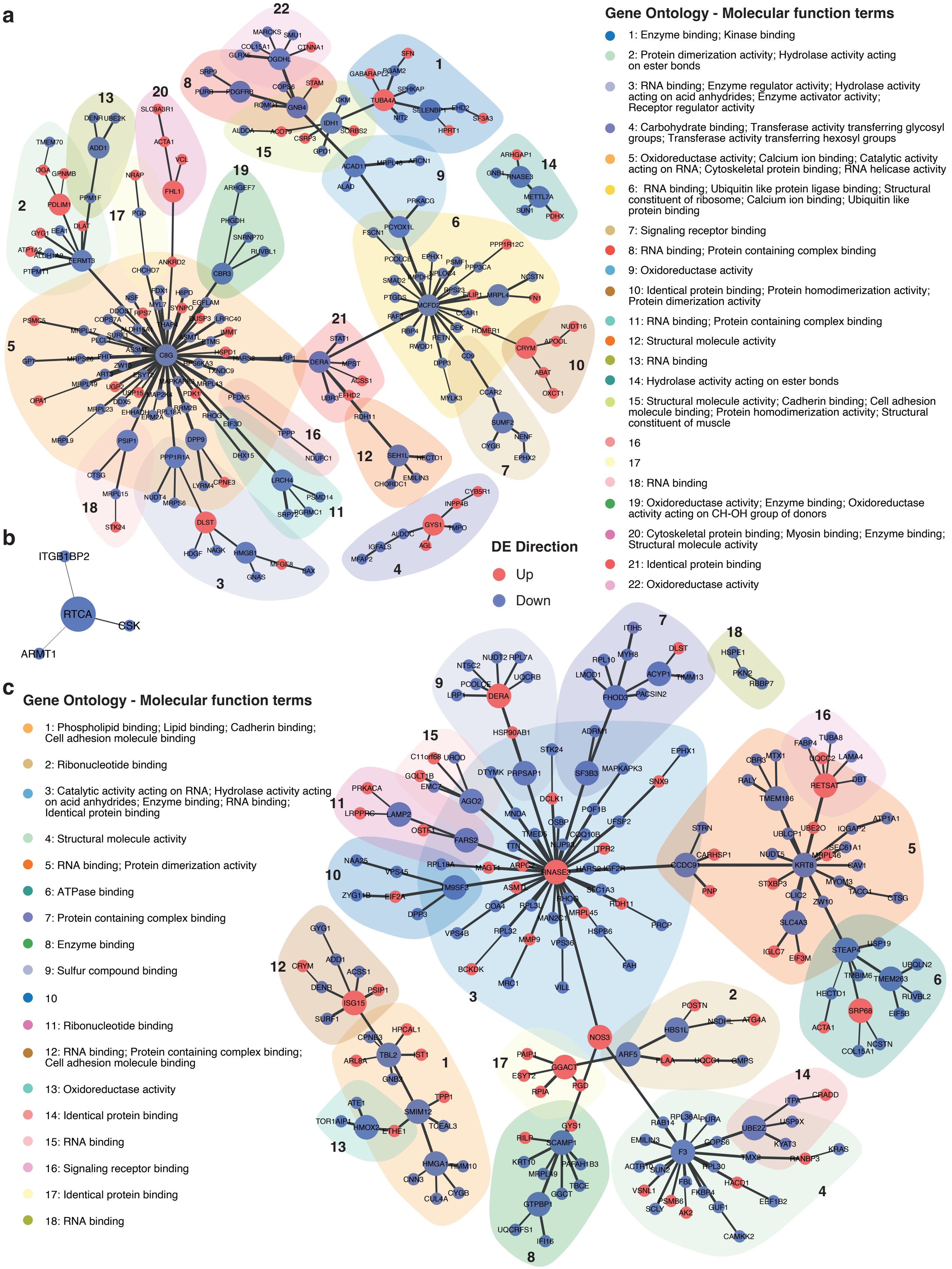

Supplementary Figure 5

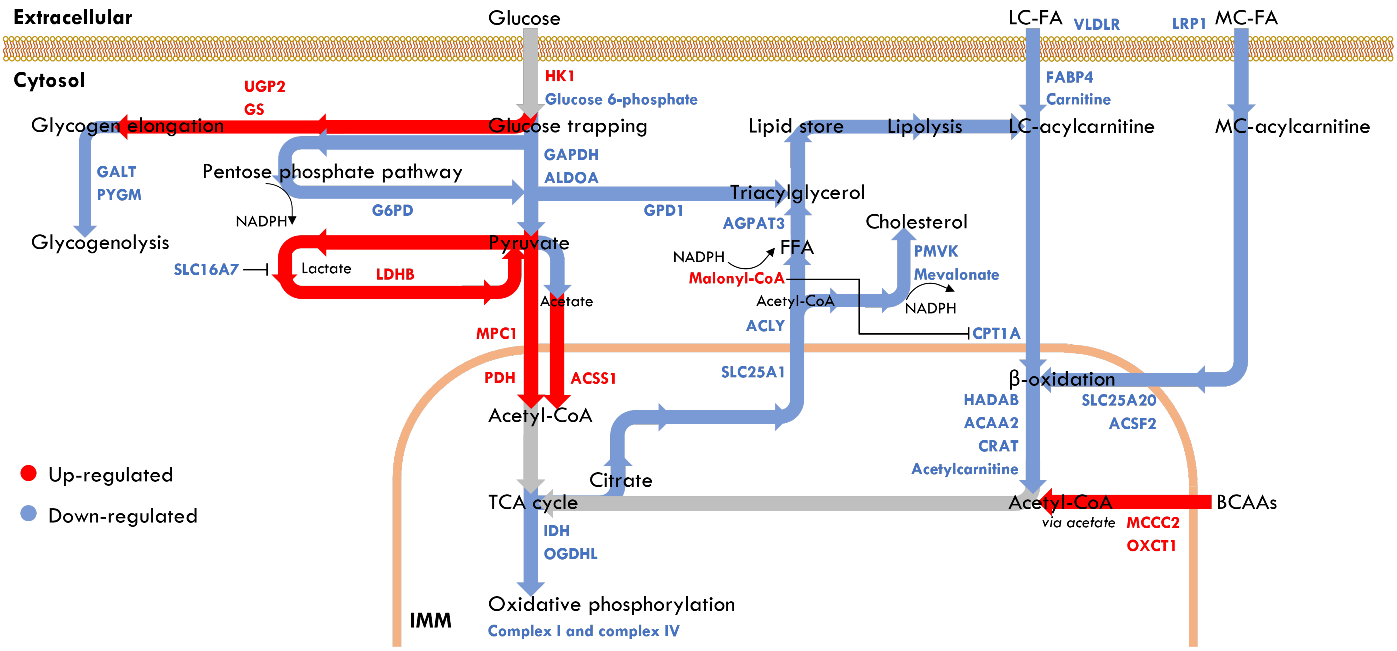

Supplement: Supplementary file 2 — Supplementary Information [file 42003_2024_7306_MOESM2_ESM.pdf]
