# Supplementary material for: Proteomic and metabolomic analyses of the human adult myocardium reveal ventricle-specific regulation in end-stage cardiomyopathies
Source: Commun Biol. 2024 Dec 19;7:1666. doi: 10.1038/s42003-024-07306-y (PMC11659555; doi:10.1038/s42003-024-07306-y)
Supplement: Supplementary file 3 — Description of Additional Supplementary File [file 42003_2024_7306_MOESM3_ESM.pdf]

Description Of Additional Supplementary File

File name: Supplementary Data 1
Description: Proteomics data log2 transformed and normalized

File name: Supplementary Data 2
Description: Proteomics sample metadata

File name: Supplementary Data 3
Description: Proteomic donor LV vs RV differential expression analysis

File name: Supplementary Data 4
Description: Proteomic KEGG pathway analysis from donor LV vs RV differential expression results

File name: Supplementary Data 5
Description: Proteomic donor LV male vs female differential expression analysis

File name: Supplementary Data 6
Description: Proteomic donor RV male vs female differential expression analysis

File name: Supplementary Data 7
Description: Proteomic heart failure (ICM and DCM combined) LV vs RV differential expression analysis

File name: Supplementary Data 8
Description: Proteomic LV vs RV differential expression result comparision between donor and heart failure (ICM and DCM combined) conditions

File name: Supplementary Data 9
Description: Proteomic non-ischaemic dilated cardiomyopathy (DCM) LV vs RV differential expression analysis

File name: Supplementary Data 10
Description: Proteomic LV vs RV differential expression result comparision between donor and DCM conditions

File name: Supplementary Data 11
Description: Proteomic ischaemic cardiomyopathy (ICM) LV vs RV differential expression analysis

File name: Supplementary Data 12
Description: Proteomic KEGG pathway analysis from ICM LV vs RV differential expression results

File name: Supplementary Data 13
Description: Proteomic LV vs RV differential expression result comparision between donor and ICM conditions

File name: Supplementary Data 14
Description: Metabolomics data log2 transformed and normalised

File name: Supplementary Data 15
Description: Metabolomics sample metadata

File name: Supplementary Data 16
Description: Metabolomic donor LV vs RV differential abundance analysis

File name: Supplementary Data 17
Description: Metabolomic KEGG pathway analysis from donor LV vs RV differential abundance results

File name: Supplementary Data 18
Description: Metabolomic donor LV male vs female differential abundance analysis

File name: Supplementary Data 19
Description: Metabolomic donor RV male vs female differential abundance analysis

File name: Supplementary Data 20
Description: Metabolomic heart failure (ICM and DCM combined) LV vs RV differential abundance analysis

File name: Supplementary Data 21
Description: Metabolomic LV vs RV differential abundance result comparision between donor and heart failure (ICM and DCM combined) conditions

File name: Supplementary Data 22
Description: Metabolomic non-ischaemic dilated cardiomyopathy (DCM) LV vs RV differential abundance analysis

File name: Supplementary Data 23
Description: Metabolomic LV vs RV differential abundance result comparision between donor and DCM conditions

File name: Supplementary Data 24
Description: Metabolomic ischaemic cardiomyopathy (ICM) LV vs RV differential expression analysis

File name: Supplementary Data 25
Description: Metabolomic LV vs RV differential abundance result comparision between donor and ICM conditions

File name: Supplementary Data 26
Description: Proteomic raw meta data for analysis

File name: Supplementary Data 27
Description: Proteomic raw sample ID to merged ID and indivdual demographic data for analysis

File name: Supplementary Data 28
Description: Metabolomic raw meta data for analysis

File name: Supplementary Data 29
Description: Metabolomic raw sample ID to merged ID and indivdual demographic data for analysis

File name: Supplementary Data 30
Description: Proteomic 2018 MS raw data IDs and link to raw data files from PRIDE
